# Effectiveness of a Brief Psychotherapeutic Intervention for Employees With Psychosomatic and Psychosocial Complaints—Pilot Study of a Consultation Off the Workplace

**DOI:** 10.3389/fpsyt.2020.00867

**Published:** 2020-09-08

**Authors:** Christine Allwang, Birgitt Marten-Mittag, Andreas Dinkel, Daniel Mauss, Claas Lahmann

**Affiliations:** ^1^ Department of Psychosomatic Medicine and Psychotherapy, Klinikum rechts der Isar, School of Medicine, Technical University of Munich, Munich, Germany; ^2^ Medical Faculty Mannheim, Mannheim Institute of Public Health, Social and Preventive Medicine, Heidelberg University, Heidelberg, Germany; ^3^ Department of Psychosomatic Medicine and Psychotherapy, University Medical Center Freiburg, Freiburg, Germany

**Keywords:** work-stress, psychotherapeutic intervention, mental health, occupational health, patient satisfaction

## Abstract

Employees’ mental health impairments are a leading reason for sickness-leave and early retirement. This is why a large number of different intervention programmes have evolved in recent years with the aim of counteracting this development. Our study evaluates a short-term cognitive-behavioral psychotherapeutic intervention off the workplace. We investigated improvement of mental and physical health in psychologically strained employees of a white collar company. Depressive symptoms (PHQ-9), anxiety symptoms (GAD-7), somatic symptoms (PHQ-15), and perceived stress (PSQ-20) were assessed at the beginning and after the intervention. Patient satisfaction (recommendation - likeliness) was also measured after the intervention. In a second step, we have looked at potential determinants of therapy outcome. Changes in the symptom measures were assessed using t-tests, MANOVA, and Chi²-tests. Cohen’s d was computed as effect size measure. One-hundred twenty-seven participants completed the assessment before, and 66 participants post intervention. Mean age of the participants was 44.6 (SD = 9.8) years, 54% were men. 89.7% of the patients attended one to five sessions. Depressive, anxiety, somatic symptoms, and perceived stress significantly declined from baseline to end of intervention. Effect sizes ranged from d = 0.49 (perceived stress) to d = 0.72 (depressive symptoms). Moreover, 93% of the patients stated that they were satisfied with the intervention and would recommend it to a friend. Previous uptake of psychiatric/psychotherapeutic treatment moderated the effect of the intervention on depressive symptoms, i.e., patients without previous experience showed a stronger reduction in symptoms of depression. The results tentatively suggest that the intervention is effective in reducing a broad range of psychological symptoms. Future research could investigate preferences and different outcomes of on-site and off-site work place interventions.

## Introduction

Mental health impairments are a relevant problem in an occupational health context and are one of the leading reasons for sickness-leave and early retirement in developed countries (1–5). In Germany, mental disorders accounted for 17% of sickness-leave days in 2014 (6), and 42% of early retirement is due to a mental health diagnosis (7).

The two most prevalent mental health diagnoses are depression and anxiety, followed by somatoform disorders (8–10). Somatoform disorders, not only predominantly pain but also other bodily symptoms, occur in the vast majority of the working population. Between 10 and 20% of employees reported to suffer from high levels of pain (11–13). These are major concern for all parties involved, as the chronic impairment at work does not only means a direct impact for the person affected but also implies a tremendous effect on the productivity of the companies, the compensation authorities, and the social welfare system (1, 5).

Although the prevalence of mental disorders is high, there is still an enormous and alarming discrepancy between demand and utilisation of mental health services (14). The consequent treatment gap is estimated between 10 and 50% (15, 16). Two prominent reasons for this are the fear of stigmatization (17) and the difficulty to access appropriate treatment options (18, 19). Fear of stigmatization withholds people to start seeing a psychotherapist at all or leads to a prolonged decision process to do so. This seems to be more prevalent in men than in women (20–22). Another reason is the difficulty and effort to access applicable therapies quickly due to long waiting periods. In Germany, the health care system covers outpatient and inpatient psychotherapy, which is provided by specialists in psychosomatic medicine, by specialists in psychiatry, or by clinical psychologists who qualify as licensed psychotherapist. Almost 100% of the German population is covered by a health insurance, which principally provides access to treatment (23). Thus, outpatient psychotherapeutic treatment should be easily available and accessible. Despite this, the average waiting time to start psychotherapy is 4.6 months, and the waiting time for a first consultation 1.9 months (19).

In light of the high prevalence of mental health problems and their impact on the affected persons and consequently on the companies, it seems to be a plausible step to address this problem in the workplace. The workplace has been identified as a suitable place to both identify mental impairments and also to initiate support at an early stage (24, 25). Therefore, some German companies started to implement a psychosomatic consultation opportunity for their employees with the idea to bypass long waiting times in the ambulatory system and to prevent long absence periods or early retirement (26).

The current common model is a so-called psychosomatic consultation in the workplace (PSIW), where employees see a specialist for mental disorders on the company grounds usually embedded in the occupational medicine clinic. Models of PSIW are already in use, scientifically evaluated, and show effective improvement of employee’s clinical and functional status (26, 27). This established model has not only a number of advantages but also some disadvantages. On one hand, it enables participants to easily access the mental health specialist on site without significant loss of travel time, the closeness to the occupational health department may create a familiar feeling and makes the first step in reaching out for help more easy. However, on the other hand, the proximity to colleagues and supervisors could also mean a disadvantage with regard to stigmatization fear. Persons who consider seeking help might be afraid as regards anonymity and a potential discrimination that may arise (17).

Our program differs from the PSIW first and foremost in terms of location, as participants see the specialist away from their workplace. A second significant variation and advantage to the referral in regular outpatient mental health care is that participants receive an appointment within only 2 weeks after they have called the clinic. Thirdly, even if the program is financed by the employer, there is strict confidentiality and anonymity toward the employer. This means in particular that the employer will not be informed whether an employee has joined the program or not, and no findings or contents of sessions are exchanged.

This pilot study investigates the effectiveness of such a short-term psychotherapeutic intervention for strained employees off the company grounds. We expected that this intervention would improve depressive, anxiety, and somatic symptoms and the level of perceived stress. Furthermore, we tried to identify determinants that could potentially have an impact on therapy outcome.

## Methods

### Design

Study participants were strained employees of an international company. Due to legal restrictions of the company involved, it was not possible to conduct a randomized controlled trial. Enrolment in the study took place consecutively between February 2014 and February 2017. The study was approved by the ethics committee of the Technical University of Munich. As this was a study within the clinical routine practice, there were no definite exclusion nor inclusion criteria. Every person referred by the company was seen and had at least one appointment with a mental health specialist. Outcome parameters, which were measures of psychological and bodily distress, were assessed immediately before the intervention (t1) and following the last intervention session (t2). In addition, patient satisfaction was measured post intervention.

### Procedure

Participants in the intervention program were employees of an international company who attended either the company’s occupational health physician or the social service department. Attendance to these services was initiated through the participants themselves or *via* the direct supervisor. Supervisors recommended a consultation with the social service department or company physician when an employee started to have noticeable problems at work, e.g., lower productivity, high error rate, or increasing number of sick-leave days.

With a referral letter of the social service department or the company’s occupational health physician, participants scheduled an appointment with our outpatient clinic themselves. The first appointment generally took place within 2 weeks depending on availability of participant and therapist, and the following sessions were scheduled individually. Participants completed self-report questionnaires upon the first appointment (t1) and following the last intervention session (t2). The post intervention assessment was sent out *via* mail. If there was no reply to the questionnaires, a reminder call was made 2 weeks later. During the initial appointment, a clinical interview and a comprehensive problem analysis were conducted. Patients were informed that the intervention comprised one to five sessions and that it would be possible to extend the intervention to a sum of 10 sessions if both the patient and the clinician would decide that this would be useful.

Participants were informed that questionnaire data will only be analyzed anonymously on a group basis. In case participants did not want to fill in the questionnaires, it had no impact on the participation in the intervention program. Participants could withdraw consent at any time for analyzing their data without any effect on their treatment in the program.

The intervention was conducted by six experienced clinicians who had at least 5 years of clinical training. Clinicians were physicians with a special training in psychosomatic medicine and psychotherapy and by clinical psychologists. They all applied cognitive behavioural interventions. The therapy content was not limited to work related problems, but attending employees could address all kinds of problems they currently saw as relevant.

### Measures

Psychological and somatic symptoms were assessed by self-report questionnaires, with modules of the patient health questionnaire (PHQ) (28) and a short version of the perceived stress questionnaire (PSQ-20).

The PHQ is widely used in different health care settings and is well-validated for clinical settings (29–31) and within the occupational field (32). Besides these questionnaires, we also assessed sociodemographic data including previous therapeutic experience, meaning previous psychosomatic or psychiatric therapeutic experience either outpatient or inpatient treatment (see **Table 1**).

**Table 1:** Sociodemographic and clinical characteristics of the study sample.

	Study group n = 127		Cases post intervention n = 66		Missing postintervention n = 61		p
	M	SD	M	SD	M	SD	
**Age (years)** Range	44.6 18-65	9.8	47.1 18-65	8.4	41.9 20-62	10.5	0.003
	N	%	n	%	n	%	
**Gender**							
Female	58	46	23	35	35	57	0.011
Male	69	54	43	65	26	43	
**Current Relationship**							
No	32	27	14	23	18	31	0.295
Yes	88	73	48	67	40	69	
**Family status**							
Single	49	40	19	29	30	51	0.059
Married	60	48	38	59	22	37	
Divorced	14	11	7	11	7	12	
Widowed	1	1	1	1	0	0	
**Children**							
Yes	61	50	34	53	27	46	0.415
**Educational level**							
Secondary school	17	13	8	12	9	15	0.915
Middle school	35	28	18	28	17	28	
Higher school certificate	22	18	12	18	10	17	
University degree	51	41	27	42	24	40	
**Employment status**							
Full time	94	76	51	80	43	73	0.687
Part time	24	20	10	16	14	24	
In training	2	2	1	2	1	2	
Miscellaneous	3	2	2	3	1	2	
**Current sickness leave**							
Yes	40	55	22	33	18	30	0.174
≤2 weeks	18	45	8	36	10	56	
>2 weeks	15	38	9	41	7	39	
No information on duration	7	18	5	23	1	6	
**Previous therapeutic experience**							
Yes	64	53	32	50	32	55	0.568
	M	SD	M	SD	M	SD	
**Number of intervention sessions**	2.9	2.3	3.7	2.7	2.1	1.4	<0.001
**Anxiety**	9.4	5.2	8.9	4.9	10.0	5.4	0.253
**Depression**	11.3	5.7	11.2	5.5	11.3	5.9	0.899
**Somatic symptoms**	10.4	6.08	10.9	6.5	9.9	5.6	0.393
**Perceived stress**	58.5	19.9	58.1	22.9	58.9	17.0	0.862

Depressive symptoms were captured by the module PHQ-9. This measure comprises nine items for screening, diagnosing, monitoring, and measuring the severity of depressive symptoms during the previous 2 weeks. The questionnaire covers the frequency of the symptoms which is rated on a four-point scale from 0 (not at all) to 3 (nearly every day). The items are summed, and a score of 10 is regarded as cut-off for a clinically relevant level of depressive symptoms. Reliability in our sample was high with Cronbach’s alpha = 0.83.

Anxiety symptoms were assessed with the generalized anxiety disorder questionnaire (GAD-7), also a module of the PHQ. This is a seven-item questionnaire designed to assess the severity of anxiety symptoms during the previous 2 weeks (33, 34). The items are rated on a four-point scale ranging from 0 (not at all) to 3 (nearly every day). A summary score of 10 represents a cut-off for clinical levels of anxiety. The scale showed high reliability in our sample (Cronbach’s alpha = 0.87).

The module PHQ-15 assesses 15 common bodily symptoms. Participants were asked to rate their severity in the past 4 weeks on a three-point scale from 0 (not bothered at all) to 2 (bothered a lot). As with the other two PHQ modules, a summary score of 10 indicates clinical levels of impairment due to bodily symptoms. Again, internal consistency was high with Cronbach’s alpha = 0.76.

Perceived stress was measured with the short version of the PSQ-20, an instrument to assess subjectively experienced stress independent of a specific and objective occasion (35). The items were rated on a four-point scale from 1 (almost never) to 4 (usually). Cronbach’s alpha in our study population was 0.91.

We also surveyed patient satisfaction with the intervention program post intervention using a ten point scale of recommendation likeliness (0 = very unlikely to 10 = very likely).

### Statistical Analysis

Data for sample characteristics are presented as absolute and relative frequencies or means and standard deviations. Mean differences were analyzed using t-tests, and proportions of categorical variables were compared using Chi²-tests. Paired sample t-tests were used to investigate changes in depression, anxiety, somatic symptoms, and perceived stress from baseline to post treatment. Correlations of depression, anxiety, somatic symptoms, and perceived stress at baseline with number of treatment sessions were reported as Pearson’s correlations coefficients r.

Repeated measures analyses of variance (MANOVA) were performed with time as the within-subject factor and one of the following categories as between-subjects factor: age (≤47 vs. >47 years), gender, number of sessions, and previous psychotherapeutic experience. Effect sizes (ESs) were computed for the pre to post changes of all outcome variables. ESs for time effects were reported by means of Cohen’s d for all outcomes (d ≤ 0.2 small effect; d = 0.5 medium effect; d ≥ 0.8 large effect). Changes in prevalence rates between baseline and post intervention were analyzed by McNemar tests. All statistical tests were two tailed. Results of p < 0.05 were regarded as statistically significant. All analyses were conducted using SPSS 22.0.

## Results

An overall number of n = 133 participants was included in the intervention program. Due to non-completion of the assessment measures, n = 6 participants (5, no data at pre-intervention; 1, not fluent in German) had to be excluded from the analyses.

Thus, n = 127 participants completed the initial assessment, n = 61 (48.0%) participants missed to fill in the t2 assessment, so n = 66 participants (52.0%) could be included in the pre-post analysis. Due to a technical mishap, the PSQ data were only available for n = 78 participants at baseline.

The participants who failed to fill in the post-intervention assessment did not differ significantly from those who filled in the second assessment except for age, sex, and number of sessions attended (see **Table 1**).

Participants were between 18 and 65 years of age, average age was 44.6 years (SD = 9.8), 54% of participants were male. Any history of outpatient psychotherapy had 45 (35.4%) out of the 127 participants. Nineteen (15.0%) of the participants reported previous inpatient psychosomatic or psychiatric treatment. Nine participants (7.1%) were in current outpatient psychotherapy, and n = 4 (3.1%) participants were in outpatient psychiatric care at the time of intervention. In summary, n = 64 (50.4%) participants had any kind of psychotherapeutic/psychiatric experience.

Sixty-nine participants (54.8%) made use of only one to two intervention sessions, another 44 (34.9%) took three to five sessions, and n = 13 (10.3%) took 6–10 sessions. The mean number of sessions attended in our treatment program was 2.9 (SD = 2.3) (see **Table 1**). There was no significant correlation of the number of treatment sessions with somatic symptom burden (r = 0.11, p = 0.246), with depressive (r = -0.01, p = 0.935) and anxiety (r = 0.04, p = 0.624) symptoms and perceived stress (r = 0.04, p = 0.710). At baseline depression, anxiety, somatic symptoms, and perceived stress did not differ according to gender, age, relationship status, and previous therapeutic experience (**Table 2**).

**Table 2:** Means (M) and standard deviations (SDs) of depression (PHQ), anxiety (GAD), and somatic symptoms (PHQ) and perceived stress (PSQ) at baseline for the total sample and stratified by gender, age groups, relationship status, and previous therapeutic experience.

	Depression		Anxiety		Somatic symptoms		Perceived stress	
	n = 126		n = 126		n = 122		n = 78	
	M	SD	M	SD	M	SD	M	SD
**Total sample**	11.27	5.68	9.43	5.17	10.42	6.08	58.52	19.90
**Gender**								
Male	11.61	5.88	9.33	5.29	10.15	6.52	60.94	20.88
Female	10.85	5.46	9.55	5.06	10.77	5.51	55.26	18.67
**Age**								
≤ 47 years	11.84	5.65	10.17	5.50	11.06	6.46	61.41	16.09
≥ 48 years	10.47	5.68	8.41	4.53	9.54	5.44	54.38	24.04
**Relationship status**								
No partner	12.21	5.91	8.48	4.87	10.65	6.67	57.96	22.50
Partner	11.23	5.67	9.90	5.18	10.61	6.02	59.93	19.21
**Previous therapeutic experience**								
No	11.55	5.53	9.14	5.14	10.30	5.74	61.75	17.98
Yes	11.39	5.79	10.05	5.10	11.08	6.29	57.55	19.84

None of the scale means differs significantly between the groups.

**Figure 1:**
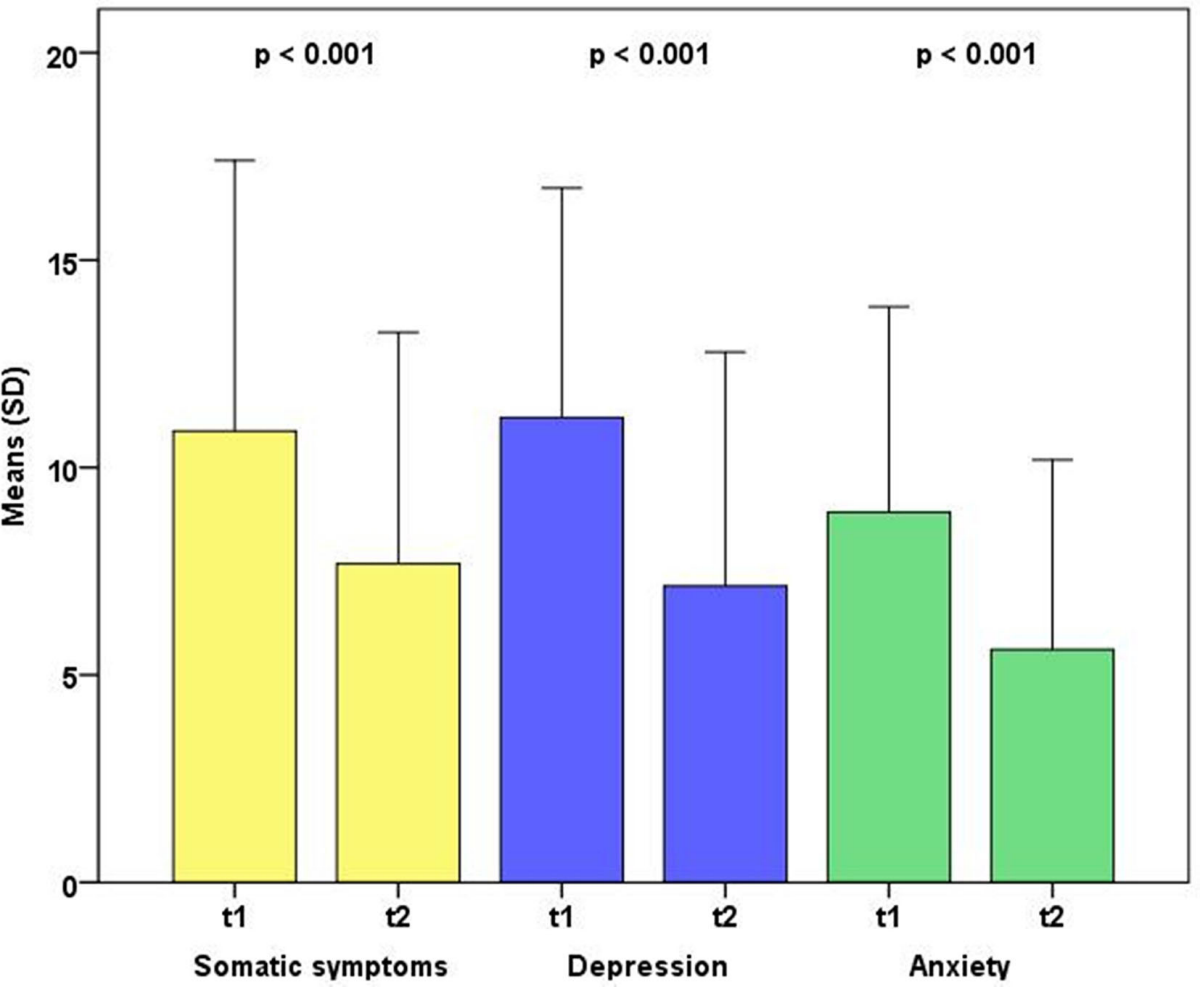
Changes in somatic, depressive, and anxiety symptoms from baseline to follow-up (n = 62–65).

We found a significant reduction in the level of depression, anxiety, somatic symptoms, and perceived stress from baseline to end of intervention (**Figure 1**). Depression dropped from M = 11.2 (SD = 5.6) to M = 7.2 (SD = 5.7); p < 0.001; ES = 0.72. Anxiety declined from M = 8.8 (SD = 4.9) to M = 5.6 (SD = 4.6); p < 0.001; ES = 0.68. Somatic symptoms decreased from M = 11.0 (SD = 6.5) to 7.7 (SD = 5.7); p < 0.001; ES = 0.54. Finally, perceived stress fell from M = 55.8 (SD = 22.9) at baseline to M = 44.1 (SD = 25.1) at end of intervention; p < 0.001; ES = 0.49.

In a second step, we investigated the change in prevalence rates for psychological and somatic symptoms. The prevalence at baseline ranged from 44.6% for anxiety to 54.8% for severe somatic symptoms. We found a significant decrease in prevalence rates from baseline to end of intervention in depression, anxiety and somatic symptoms (**Table 3**). Due to the lack of an agreed-upon cut-off score, this analysis was not performed on the PSQ.

**Table 3:** Prevalence rates (%) of clinical levels of somatic symptoms, depression (PHQ) and anxiety (GAD) at baseline and end of intervention.

	n	Baseline	End of intervention	p
**Severe somatic** **symptoms**	62	54.8	29.0	<0.001
**Depression**	64	54.7	28.1	0.001
**Anxiety**	65	44.6	20.0	<0.001

McNemar test; exact significance two-sided.

For exploratory reasons, we investigated whether gender and previous experience with psychiatric/psychotherapeutic treatment exerted a moderating effect on the outcome variables. Due to the low number of available data, this analysis was not performed for perceived stress.

**Figure 2:**
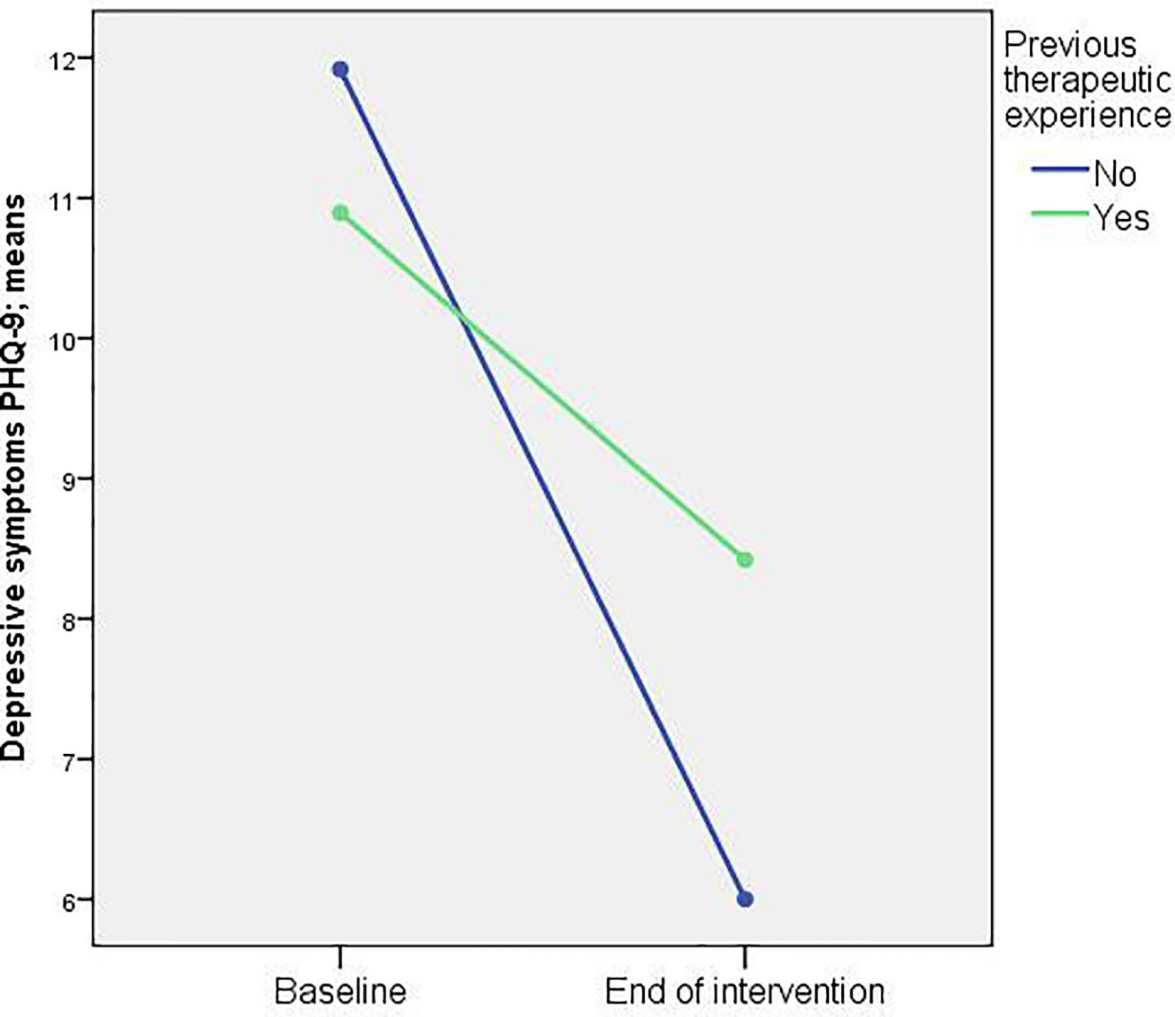
Changes in depressive symptoms stratified by previous psychotherapeutic/psychiatric experience (n = 62).

We found only one significant result. A significant interaction of time by previous therapy experience emerged for depressive symptoms (p = 0.018). Participants without previous therapeutic experience showed a stronger reduction of mean depression score at the end of the intervention [from M = 11.9 (SD = 5.7) to M = 6.0 (SD = 4.7)] than participants with previous use of therapies [from M = 10.9 (SD = 5.3) to M = 8.4 (SD = 6.3)] (**Figure 2**).

Regarding patient satisfaction the responses of n = 56 (84.8%) were available.

The results showed that 52 participants (92.9%) would recommend the program to a friend (recommendations score ≥ 8).

## Discussion

Mental health problems in the occupational context are problematic not only for the affected employees but also for the companies being concerned with presenteeism, long times of sickness absence, and early retirement eventually. Therefore, it seems to be a plausible step for companies to prevent a lack of performance due to presenteeism or imminent absences and to create a professional setting for strained employees to address their problems with a specialist.

Our study examined a novel approach where employees got the opportunity to see a mental health specialist off site within a narrow time frame. The main finding of our study was a significant reduction of depressive, anxiety, and somatic symptoms and perceived stress from the beginning to the end of the intervention sessions, with moderate to large effect sizes. Our results support findings seen in other studies showing that a short-term psychotherapeutic intervention can improve the wellbeing of employees and support their psychological health in a positive way (26, 27).

Rothermund et al. (26) showed also that the workplace is a well-suited place to identify persons at risk for developing mental health problems who require treatment and to introduce them quickly to professional mental health services (26). This is highly important as even mild depressive symptoms result in reduced work productivity (36); on top of that, it is well known that mental health disorders are best treated in their early stages to prevent chronification that implies the risk of long-term absence from work or even early retirement (37–39).

We obtained one interaction effect. Participants without previous therapeutic experience had a significantly higher benefit out of the intervention program than participants who had any kind of previous psychiatric/psychotherapeutic treatment. This might be due to a more chronic course of psychosocial problems in patients with previous use of therapy, indicating that a short-term intervention might be less effective in patients with long-lasting symptoms.

The vast majority of participants (93%) was satisfied with the intervention program and would recommend it to a friend. This is a clearly positive criterion for the feasibility and quality of our intervention program.

Employees who attended our short-term intervention program were guaranteed to get a quick appointment with a specialist of mental health care. The service offered is located off the company grounds and participation is absolutely anonymous to the employer. These two factors could be important in the decision making process to venture into the program. Especially, the option to see a mental health specialist away from the company’s premises and the easy access to our program seems to be a potential factor that reduces the inhibition threshold toward psychotherapy in general but in particular in men and their stigmatization fear.

This could be one reason for the high rate of men in our study. Fiftyfour percent (54%) of participants were male, which is different from regular outpatient psychotherapy. Here, men account for about 25–30% of attendees (22, 40). Studies show that the male population has still a very sceptic attitude toward psychotherapy, whereas females are more open to begin psychotherapy (41).

In future studies it could be of interest to look further into the question if there are certain characteristics that influence the preference of therapy location, meaning whether an employee prefers an on-site or off-site consultation.

Clearly, there are some limitations to our study. First, we used only self-report measures and did not apply a structured clinical interview for diagnosing mental disorders. Second, the response rate at the end of intervention was low, which limits the significance of our results. Third, we did not have a control group as this was an employer funded program and which did not allow to apply a randomized controlled trial or a wait-list design. Fourth, a “healthy worker effect” could have led to a selection bias and underestimation of effect sizes, meaning that employees with severe health complains have been sick at home or had already left the company and therefore did not participate in this study. Finally, only individuals who presented to a company-physician or the social service department were included.

In conclusion, our program is a quick and easy access for strained employees to mental health care specialists at an early stage of mental distress. Furthermore, the results of this off-site intervention tentatively suggest that it constitutes an effective and well-accepted way of alleviating symptom burden, and it seems to be especially attractive to male employees.

## Data Availability Statement

The datasets generated for this study are available on request to the corresponding author.

## Ethics Statement

The studies involving human participants were reviewed and approved by Ethikkommission an der Technischen Universität München. Written informed consent for participation was not required for this study in accordance with the national legislation and the institutional requirements.

## Author Contributions

CA and CL designed and planed the study. CA conducted the study. The data was analyzed by CA, BM-M, and AD, and the first draft was written by CA. All authors contributed to the article and approved the submitted version.

## Funding

The publication of this work as an open access article will be supported by the German Research Foundation (DFG) and the Technical University of Munich (TUM) within the funding programme Open Access Publishing.

## Conflict of Interest

The authors declare that the research was conducted in the absence of any commercial or financial relationships that could be construed as a potential conflict of interest.
